# Computational Investigation of an All-*sp*^3^ Hybridized Superstable Carbon Allotrope with Large Band Gap

**DOI:** 10.3390/ma18112533

**Published:** 2025-05-28

**Authors:** Xiaoshi Ju, Kun Bu, Chunxiao Zhang, Yuping Sun

**Affiliations:** School of Physics and Optoelectronic Engineering, Shandong University of Technology, Zibo 255000, China; juxiaos@163.com

**Keywords:** carbon, first-principles calculations, X-ray diffraction

## Abstract

Carbon is one of nature’s basic elements, hosting a tremendous number of allotropes benefiting from its capacity to generate sp, $sp^{2}$, and $sp^{3}$ hybridized carbon–carbon bonds. The exploration of novel carbon architectures has remained a pivotal focus in the fields of condensed matter physics and materials science for an extended period. In this paper, we, by using first-principles calculation, carry on a detailed investigation an an all-$sp^{3}$ hybridized carbon structure in a 20-atom tetragonal unit cell with $P4_{3}2_{1}2$ symmetry ($D_{4}^{8}$, space group No. 96), and call it T20 carbon. The equilibrium energy of T20 carbon is −8.881 eV/atom, only 0.137 eV/atom higher than that of diamond, indicating that T20 is a superstable carbon structure. T20 is also a superhard carbon structure with a large Vicker’s hardness about 83.5 GPa. The dynamical stability of T20 was verified by means of phonon band spectrum calculations. Meanwhile, its thermal stability up to 1000 K was verified via ab initio molecular dynamics simulations. T20 is an indirect band-gap insulator with approximately 5.80 eV of a band gap. This value is obviously greater than the value in the diamond (5.36 eV). Moreover, the simulated X-ray diffraction pattern of T20 displays a remarkable match with the experimental data found in the milled fullerene soot, evidencing that T20 may be a potential modification discovered in this experimental work. Our work has given a systematical understanding on an all-$sp^{3}$ hybridized superstable and superhard carbon allotrope with large band gap and provided a very competitive explanation for previous experimental data, which will also provide guidance for upcoming studies in theory and experiment.

## 1. Introduction

Carbon is one of nature’s basic elements, hosting a tremendous number of allotropes benefiting from its capacity to generate sp, $sp^{2}$, and $sp^{3}$ hybridized carbon–carbon bonds [1]. Multiple physical properties, such as superhardness [2], semiconductivity [3,4], and electronic band topologies [5,6,7,8,9,10,11,12,13,14,15,16,17,18,19,20], can all be realized in all-carbon materials. The pursuit of new carbon allotropes with remarkable properties is one of the central topics in condensed matter physics and materials sciences. The most prominent experimentally synthesized examples include the zero-dimensional (0D) fullerene [21], one-dimensional (1D) carbon nanotube (CNT) [22], and two-dimensional (2D) graphene [23]. The three-dimensional (3D) carbon allotropes are often explored under harsh conditions, including high pressure, high temperature, and shock compressions. For example, a BC8 [24] carbon was reported to be synthesized by compressing diamond at the high pressure about 1100 GPa [24,25,26,27]; several new hard carbon structures have been found in the cold compressed graphite at room temperature [28]; a number of emerging carbon allotropes have also been discovered in detonation [29] and chimney soot [30]; and an “*n*-diamond” (new-diamond) phase has been found in multiple experiments [31,32,33,34,35,36,37]. Experimental searches for new carbon allotropes continue, and very recently the 2D [38] and 3D fullerene networks [39] have been reported to have been synthesized.

Besides these comprehensive experimental efforts, another intriguing research direction is to explore carbon structures using multiple theoretical techniques, and a series of carbon structures has been theoretically proposed [40,41,42,43,44,45,46,47,48,49]. Nowadays, many of the theoretically proposed carbon structures have been included in the international data base SACADA for carbon allotropes [50,51]. Theoretical design can also provide guidance for experiments, for example, all-$sp^{3}$ hybridized superhard M- [45], ${\mathrm{bct-C}}_{4}$ [46], W- [47], and Z-carbon [48] have been proposed to explain the new carbon phases found in the cold compressed graphite, and a series of all-$sp^{2}$ hybridized carbon allotropes [3,8,9,19] has been proposed to explain the new carbon allotropes found in detonation [29] and chimney soot [30], and some of them hold exotic electronic band topologies [8,9,19]. Some carbon phases are firstly theoretically proposed and later synthesized in experiments, such as the T-carbon [49,52]. Theoretically, a carbon structure can be characterized as a finite Graph, the carbon atoms can be viewed as vertices, and the carbon–carbon bonds can be viewed as edges. This thought is originally from the pioneering works of A. F. Wells [53]. Recently inspired by this thought, a highly efficient structural search method $RG^{2}$ has been invented [54,55].

In this study, we present the results of first-principles calculations for a comprehensive investigation into an all-$sp^{3}$ hybridized carbon allotrope. This carbon structure has a 20-atom tetragonal unit cell with $P4_{3}2_{1}2$ symmetry ($D_{4}^{8}$, space group No. 96); thus, we call it T20 carbon. T20 carbon has a very low equilibrium energy about −8.881 eV/atom, which is only 0.137 eV/atom higher than that of diamond, indicating that T20 is a superstable carbon structure. Meanwhile, T20 is also a superhard carbon structure with a large Vicker’s hardness about 83.5 GPa. The dynamical stability of T20 was validated through phonon band spectrum calculations, while its thermal stability up to 1000 K was confirmed by ab initio molecular dynamics (AIMD) simulations. T20 is an indirect band-gap insulator with a 5.80 eV of large band gap, which is obviously greater than the value in the diamond (5.36 eV). Moreover, the simulated X-ray diffraction (XRD) pattern of T20 shows an excellent match with the experimental data found in the milled fullerene soot [56]; also, considering the super stability of T20, T20 is a very competitive explanation for this experiment. Our work has provided an in-depth understanding on this all-$sp^{3}$ hybridized superstable and superhard carbon structure with large band gap, which has enriched the family of all-$sp^{3}$ hybridized carbon allotropes and provided guidance for previous experimental data. Our results will also provide guidance for upcoming theoretical and experimental studies.

## 2. Computational Method

The T20 carbon structure can be discovered using the $RG^{2}$ code based on a graph theoretic method [54,55]. During the structural search process, the bonding types are set to be all-$sp^{3}$ hybridized, the number of carbon atoms is set to be 20, and the unit cell is set to be tetragonal. Besides the T20 carbon structure, a series of all-$sp^{3}$ hybridized carbon structures is also derived. Many of them are complicated lower-symmetry structures, which are not of direct interest to the present work and are therefore omitted. Our ab initio calculations are performed using the Vienna ab initio simulation package (VASP) [57]. To make a comparison with the previously reported oP16 carbon structure [14] and to make a complete series, the generalized gradient approximation (GGA) developed by Armiento–Mattsson (AM05) [58] is used as the exchange-correlation potential for the structural optimizations and total energy calculations. Meanwhile, we have also checked the lattice parameters using the standard GGA-PBE functional [59]. We use the all-electron projector augmented wave (PAW) [60] method, and the carbon 2$s^{2}$2$p^{2}$ electrons are taken as valence electrons. A plane-wave basis set with an energy cutoff of 800 eV is implemented. The Brillouin zone sampling is achieved through a 8 × 8 × 8 Monkhorst–Pack k-point mesh. Geometric optimizations are conducted under symmetry constraints, terminating when the residual atomic forces drop below $10^{-3}$ eV/Å. The energy convergence threshold is set to $10^{-6}$ eV. The electronic band structures are computed using the Heyd–Scuseria–Ernzerhof hybrid functional (HSE06) [61]. The phonon band structures and force constants are computed via the finite displacement method implemented in the Phonopy package [62].

## 3. Results and Discussion

**Figure 1:**
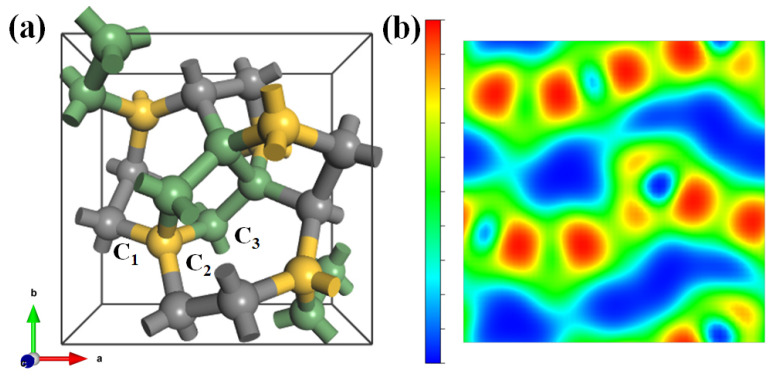
(**a**) As the unit cell of T20 carbon, T20 has an all-$sp^{3}$ hybridized tetragonal unit cell with 20 carbon atoms in $P4_{3}2_{1}2$ symmetry ($D_{4}^{8}$, space group No. 96). The equilibrium lattice parameters are a=b=5.801 Å, and c=3.441 Å. There are three kinds of carbon atoms occupying the 8b (0.1374, 0.5691, and 0.0476), 4a (0.6987, 0.6987, and 0.0), and 8b (0.0222, 0.8493, and 0.5812) Wyckoff positions, denoted as $C_{1}$ (black), $C_{2}$ (yellow), and $C_{3}$ (green), respectively. (**b**) The electron localization function (ELF) map for T20 carbon viewed along the (001) direction, the distance to the origin is set to be 3.0 Å.

The crystalline structure and electron localization function map for T20 carbon. Firstly, we analyze the crystalline structure of T20 carbon. The unit cell of T20 carbon is depicted in Figure 1a. The T20 carbon structure has an all-$sp^{3}$ hybridized tetragonal unit cell with 20 carbon atoms in $P4_{3}2_{1}2$ symmetry ($D_{4}^{8}$, space group No. 96). The equilibrium lattice parameters are a=b=5.801 Å, and c=3.441 Å. There are three kinds of carbon atoms occupying the 8b (0.1374, 0.5691, and 0.0476), 4a (0.6987, 0.6987, and 0.0), and 8b (0.0222, 0.8493, and 0.5812) Wyckoff positions, denoted as $C_{1}$, $C_{2}$, and $C_{3}$, respectively. The bond lengths in the T20 carbon range from 1.524 to 1.569 Å, and the carbon atoms form (5+7+8)-membered rings in this carbon structure. Meanwhile, we have also checked the lattice parameters using the standard GGA-PBE functional, which are a=5.8314 Å, b=5.8314 Å, and c=3.4612 Å. Figure 1b shows the electron localization function (ELF) map for T20 carbon viewed along the (001) direction; the distance to the origin is set to be 3.0 Å. It can be seen from the ELF map that the charge density is localized around the carbon atoms; then, we can further infer the all-$sp^{3}$ covalent bonding of T20 carbon.

**Figure 2:**
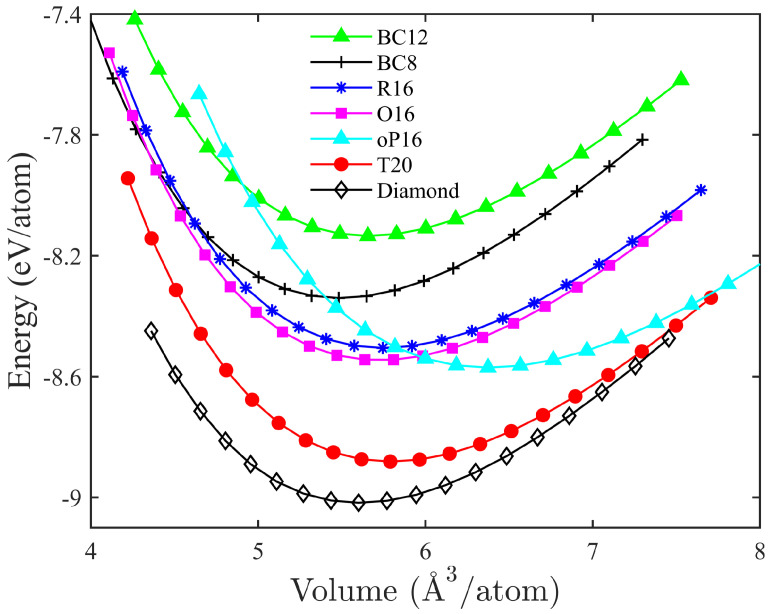
The curve of energy per atom versus volume for T20 carbon. The diamond, and previously reported all-$sp^{3}$ hybridized BC12 [41], BC8 [24,25,26,27], R16 [42], O16 [44], and $sp^{2}$-$sp^{3}$ hybridized oP16 carbon [14], are taken as a point of comparison. The equilibrium energy is about −8.881 eV/atom for T20 carbon.

Energetic and mechanical properties of T20 carbon. Figure 2 illustrates the evolution of energy per atom depending on the volume for T20 carbon, compared with diamond and the reported BC12 [41], BC8 [24,25,26,27], R16 [42], O16 [44], and oP16 carbon [14]. The equilibrium energy is about −8.881 eV/atom for T20 carbon, which is only 0.137 eV/atom higher than the equilibrium energy of diamond and lower than all the other carbon allotropes, indicating that T20 is a superstable carbon structure. By fitting the energy versus volume curve with the Murnaghan’s equation of state [63]:

(1) $$E\left(V\right)=E\left(V_{0}\right)+\frac{B_{0}V}{B_{P}}\left[\frac{{\left(V_{0}/V\right)}^{B_{P}}}{B_{P}-1}+1\right]-\frac{V_{0}B_{0}}{B_{P}-1},$$

we derive that the bulk modulus ($B_{0}$) value for T20 carbon is about 422 GPa, which is smaller than 451 GPa for diamond, and 429 GPa for BC12 carbon, but larger than 407 GPa for BC8, 386 GPa for R16, 418 GPa for O16, and 370 GPa for oP16 carbon. Moreover, to examine the mechanical stability of T20 carbon, we have calculated the elastic constants for T20 carbon as $C_{11}=1170$ GPa, $C_{12}=102$ GPa, $C_{13}=156$ GPa, $C_{33}=964$ GPa, $C_{44}=510$ GPa, and $C_{66}=560$ GPa. These values satisfy the criteria [64] of mechanical stability for the tetragonal phase as $C_{11}>0$, $C_{33}>0$, $C_{44}>0$, $C_{66}>0$, $(C_{11}-C_{12})>0$, $(C_{11}+C_{33}-2C_{13})>0$, and $[2\left(C_{11}+C_{12}\right)+C_{33}+4C_{13}]>0$. Then, using the Viogit’s scheme of averaging [64]:

(2) $$\begin{array}{cc} B_{V} & =\left(1/9\right)[2\left(C_{11}+C_{12}\right)+C_{33}+4C_{13}],\\ G_{V} & =\left(1/30\right)\left(M+3C_{11}-3C_{12}+12C_{44}+6C_{66}\right),\\ M & =C_{11}+C_{12}+2C_{33}-4C_{13}. \end{array}$$

we derive the bulk modulus $B_{V}$ and shear modulus $G_{V}$ as $B_{V}=459$ GPa and $G_{V}=509$ GPa. Moreover, according to the formula for Vicker’s hardness suggested by Chen et al. [65]:

(3) $$H_{v}=2{\left(G_{V}^{3}/B_{V}^{2}\right)}^{0.585}-3,$$

the calculated Vicker’s hardness for T20 carbon is 83.5 GPa, which is close to 85 GPa for O16 and larger than 56 GPa for oP16, 82.5 GPa for BC8, and 76.7 GPa for BC12 carbon, indicating that T20 carbon is also a superhard carbon structure. The large Vicker’s hardness of T20 can be attributed to its large mass density ($3.34\;{\mathrm{g/cm}}^{3}$) and all-$sp^{3}$ bonding. In Table 1, we present the structural parameters (including lattice parameters, volume, and bond lengths), bulk modulus $B_{0}$, Vickers hardness $H_{v}$, density ρ, and calculated band gaps $E_{g}$ for the above-mentioned carbon allotropes, also compared with the available experimental data for diamond [66].

**Figure 3:**
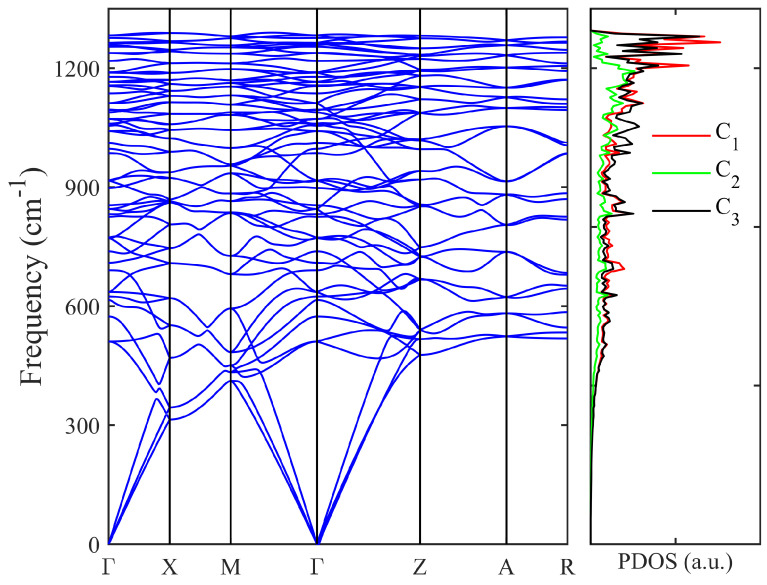
The phonon band structure and phonon density of states (PDOS). The highest phonon frequency is about $1288\;{\mathrm{cm}}^{-1}$ at the high-symmetric X point. The high phonon frequency modes are mainly contributed by the $C_{1}$ and $C_{3}$ carbon atoms. There is no imaginary frequency in the entire BZ and PDOS, thus confirming the dynamical stability of T20 carbon.

Phonon band spectrum of T20 carbon. In order to assess the dynamical stability of T20 carbon, we have computed the phonon band dispersion and phonon density of states (PDOS), which are illustrated in Figure 3. The highest phonon frequency for T20 carbon is about $1288\;{\mathrm{cm}}^{-1}$ at the high-symmetric X point. There are three acoustic phonon modes and 57 optical phonon modes in the T20 carbon structure. There is no imaginary frequency in the entire BZ and PDOS, thus confirming the dynamical stability of T20 carbon. From the PDOS calculations, we can see that the high-frequency phonon modes are mainly contributed by the $C_{1}$ and $C_{3}$ carbon atoms since there are eight $C_{1}$ and eight $C_{2}$ carbon atoms, while the $C_{2}$ carbon atoms make fewer contributions since there are only four $C_{2}$ carbon atoms.

**Figure 4:**
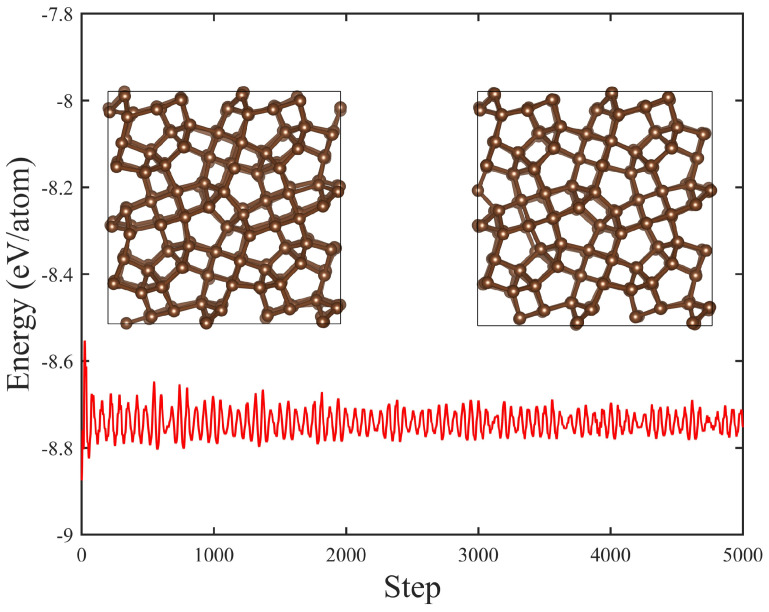
The ab initio molecular dynamics simulations (AIMD) for T20 carbon at the temperature of 1000 K for 5 ps with the time step of 1 fs. The inset structures show the crystalline structures at step 1000 and 5000 during the simulations.

Ab initio molecular dynamics of T20 carbon. To examine the thermal stability of T20 carbon, we have performed ab initio molecular dynamics (AIMD) simulations using a 2 × 2 × 3 supercell with 320 carbon atoms in total with a canonical ensemble (NEV) and a Nosé thermostat [67] and using a step of 1 fs and 5 ps in total. The energy fluctuations during the AIMD simulations are shown in Figure 4; the inset structures show the crystalline structures during the simulation step 1000 and 5000, respectively. As demonstrated, no significant structural distortions took place throughout the simulation. Certain carbon atoms might exhibit minor displacements from their original equilibrium sites, yet they are anticipated to relax to the equilibrium sites following appropriate structural refinements, signifying the excellent thermal endurance of T20 carbon up to the elevated temperature of 1000 K.

**Figure 5:**
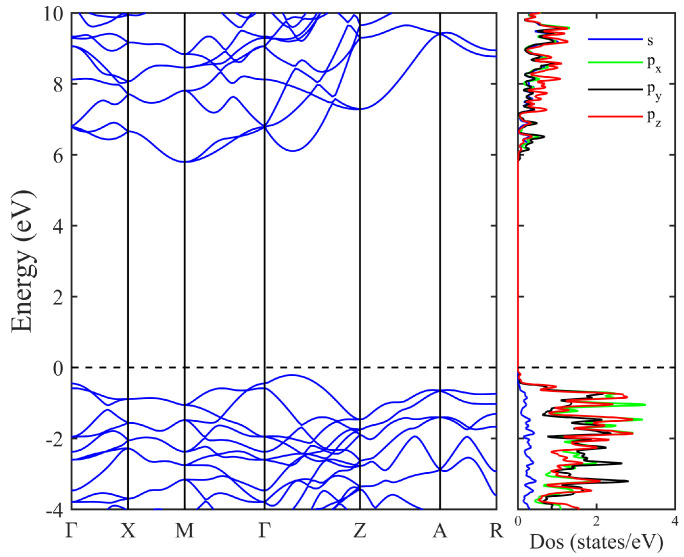
The electronic band structures and orbital projected density of states (DOS) of T20 carbon. T20 is an insulator with an indirect band gap about 5.80 eV; the conduction band minimum (CBM) is at the high-symmetric M point, and the valence band maximum (VBM) is along the high-symmetric Γ-Z path.

Electronic properties of T20 carbon. Subsequently, we have analyzed the electronic characteristics of T20 carbon. As presented in Figure 5, the computed electronic band dispersions and orbital-projected density of states (DOS) for T20 carbon are illustrated. T20 is an insulator with a large indirect band gap about 5.80 eV, which is even larger than the band gap value of diamond (5.36 eV). The conduction band minimum (CBM) is at the high-symmetric M point, and the valence band maximum (VBM) is along the high-symmetric Γ-Z path. From the DOS calculations, we can see that the carbon $p_{x}$, $p_{y}$, and $p_{z}$ orbitals make near equal contributions to the electronic states around the Fermi level ($E_{F}$) due to the all-$sp^{3}$ bonding of T20, while the carbon *s* orbitals make fewer contributions due to the all-$sp^{3}$ bonding of T20 carbon. Meanwhile, we have also examined the band gap using the crystalline structure obtained from the standard GGA-PBE method; the calculated band gap value is about $E_{g}=5.74$ eV, which is very close to the value of 5.80 eV using the crystalline structure generated from AM05 method.

**Figure 6:**
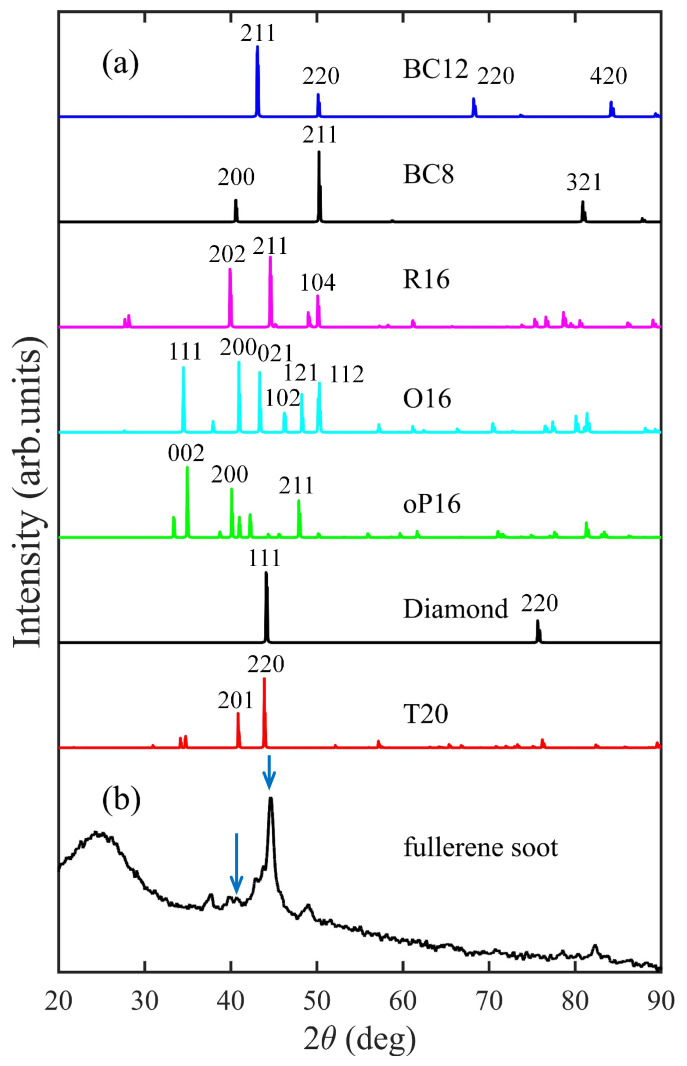
(**a**) Simulated X-ray diffraction (XRD) pattern for T20 carbon, compared with diamond, and previously reported BC12 [41], BC8 [24,25,26,27], O16 [44], R16 [42], and oP16 carbon [14], and (**b**) the experimental data derived from milled fullerene soot [56]. The X-ray wavelength is 1.5406 Å with a copper source.

X-ray diffraction pattern of T20 carbon. For the purpose of correlating with experimental results, we have simulated the X-ray diffraction (XRD) pattern of T20 carbon compared with diamond; the previously reported BC12 [41], BC8 [24,25,26,27], R16 [42], O16 [44], and oP16 carbon [14]; and the experimental data derived from the milled fullerene soot [56], as shown in Figure 6. The (201) peak at around 40.6°, and the (220) peak at around 44.1° show a good match with some of the peaks found in this experiment, indicating that T20 may be one of the modifications found in this experiment. Previously, the R16 carbon was proposed to explain this experiment; however, the equilibrium energy of T20 carbon is a lot lower than that of R16, showing that T20 is a more competitive explanation for this experiment.

## 4. Summary

In summary, we have performed a systematical ab initio study on an all-$sp^{3}$ hybridized T20 carbon and focused on its structural, energetic, mechanical, and electronic properties. T20 carbon is a superstable carbon structure with equilibrium energy only 0.137 eV/atom higher than that of diamond, and a superhard carbon structure with a large Vicker’s hardness about 83.5 GPa. The phonon band spectrum calculations and ab initio molecular dynamics simulations have, respectively, validated the dynamical and thermal stabilities of T20 carbon. The calculated electronic band structures indicate that T20 is an insulator featuring a 5.80 eV of an indirect band gap. Moreover, the simulated X-ray diffraction pattern of T20 carbon shows a good match with the experimental data found in the milled fullerene soot, which makes T20 a very competitive explanation for this experiment due to its super energetic stability. Our work has identified a novel superstable and superhard carbon structure, which has enriched the family of all-$sp^{3}$ hybridized carbon allotropes and will provide guidance for future theoretical and experimental studies in related fields.

## Figures and Tables

**Table 1:** Computed equilibrium structural parameters (space group; lattice parameters *a*, *b*, and *c*; angle α; volume per atom $V_{0}$; and bond lengths $d_{C-C}$); total energy $E_{tot}$ per atom; electronic band gap $E_{g}$; bulk modulus $B_{0}$; Vickers hardness $H_{v}$; and density ρ for diamond, BC12 [41], BC8 [24,25,26,27], R16 [42], O16 [44], oP16 [14], and T20 carbon at zero pressure, compared to available experimental data [66].

Structure	Space Group	Method	*a* (Å)	*b* (Å)	*c* (Å)	γ (°)	$V_{0}$ (${Å}^{3}$)	$d_{C-C}$ (Å)	$E_{\mathrm{tot}}$ (eV)	$E_{g}$ (eV)	$B_{0}$ (GPa)	$H_{v}$ (GPa)	ρ (${\mathrm{g/cm}}^{3}$)
Diamond	$Fd\bar{3}m$	AM05	3.552				5.60	1.538	−9.018	5.36	451	93.5	3.45
		Exp [66]	3.567				5.67	1.544		5.47	446	96	3.52
BC12	$Ia\bar{3}d$	AM05 [41]	5.139				5.66	1.574	−8.134	2.98	429	76.7	3.41
BC8	$Ia\bar{3}$	AM05 [41]	4.443				5.48	1.617	−8.340	3.58	407	82.5	3.53
R16	$\bar{R}3c$	AM05	4.514			90.88	5.75	1.466–1.755	−8.505	4.45	386	91	3.36
O16	Pbcn	AM05	4.405	4.740	4.384		5.72	1.493–1.730	−8.546	4.23	418	85	3.38
oP16	Pcca	AM05	4.646	4.277	5.133		6.37	1.353–1.691	−8.570	Semimetal	370	56	3.03
T20	$P4_{3}2_{1}2$	AM05	5.801		3.441		5.79	1.524–1.569	−8.881	5.80	422	83.5	3.34

## Data Availability

The original contributions presented in this study are included in the article. Further inquiries can be directed to the corresponding authors.
